# Expression Profiling and Function Analysis Identified New Cumulus Cells-Expressed Genes and miRNAs Predictive of Oocyte Developmental Potential

**DOI:** 10.3390/cells14110791

**Published:** 2025-05-28

**Authors:** Min Zhang, Meng-Meng Wang, Fa-Li Zhang, Nan He, Ming-Jiu Luo, Shuai Gong, Fu-Yin Fu, Hong-Jie Yuan, Jie Zhang, Jing-He Tan

**Affiliations:** College of Animal Science and Veterinary Medicine, Shandong Agricultural University, Tai’an City 271018, China; mrzhangmin@163.com (M.Z.); yam2895@163.com (M.-M.W.); jackzhang410@163.com (F.-L.Z.); conanhe-1986@163.com (N.H.); luomj@sdau.edu.cn (M.-J.L.); gongshuai5@sdau.edu.cn (S.G.); 15288916369@163.com (F.-Y.F.); yuanhj@sdau.edu.cn (H.-J.Y.)

**Keywords:** cumulus cells, developmental potential, genes, miRNAs, oocytes

## Abstract

Although prior studies have identified cumulus cells (CCs)-expressed genes and miRNAs that regulate cumulus expansion and/or CC apoptosis and may serve as markers for selecting competent oocytes and embryos, there remains an urgent need to identify CCs-expressed genes and miRNAs whose expression levels are directly correlated with oocyte developmental potential (DP). In this study, we first established CC models from mouse cumulus-oocyte complexes (COCs) that exhibited significantly different DP following in vitro or in vivo maturation. Subsequently, we performed mRNA/miRNA sequencing and functional analyses using these in vitro and in vivo CC models. We identified and validated Spp1, Fn1, Sdc1, and Ngf as DP-beneficial genes; Fos and Jun as DP-detrimental genes; and miR-7686-5p, miR-133a-3p, novel-miR-239, novel-miR-193, and miR-339-5p as DP-detrimental miRNAs. Finally, by employing a well-in-well activation/embryo culture system that enables tracking the COC origin of CCs and embryos, we further validated Spp1 and Ngf as DP-beneficial genes, Jun as the DP-detrimental gene, and miR-7686-5p, novel-miR-239, and miR-339-5p as DP-detrimental miRNAs. In conclusion, we identified and validated new sets of CCs-expressed genes and miRNAs whose expression levels were directly correlated with oocyte DP. These newly identified genes and miRNAs may serve as potential biomarkers for selecting competent oocytes and embryos.

## 1. Introduction

It is widely recognized that in vitro maturation (IVM) can generate a substantial number of mature oocytes for use in assisted reproductive technologies (ART) in both humans and animals. However, the developmental potential (DP) of IVM oocytes is considerably lower than that of in vivo matured oocytes [1,2,3]. Consequently, current IVM protocols require optimization to enhance the quality of oocytes produced for ART applications. The selection of competent oocytes prior to IVM culture is critical, as numerous factors—including donor age, estrous cycle stage, nutritional status, health condition, and genetic potential—can significantly impact oocyte quality, particularly when animal ovaries sourced from slaughterhouses are utilized [4]. Moreover, selecting embryos with the highest DP for transfer is essential for successful human in vitro fertilization (IVF). Identifying the single embryo with the optimal DP in each cycle would enable elective single-embryo transfer, effectively eliminating multiple pregnancies while maximizing the pregnancy probability for each patient [5,6]. Therefore, ongoing research efforts will continue to prioritize the development of efficient evaluation frameworks for oocytes and embryos.

The current strategies for assessing oocyte quality predominantly rely on apoptotic indices of cumulus cells (CCs) [7] and the morphology of cumulus expansion [8]. However, the precision of these methods falls short of the desired level. Consequently, the development of noninvasive, objective, accurate, rapid, and cost-effective tests for pre- and post-IVM assessment of oocyte DP remains one of the most significant contemporary objectives in ART research. Given that the oocyte regulates CC function during follicle development, CC function is expected to indirectly reflect oocyte DP [9,10]. Therefore, analyzing gene expression in CCs can serve as a noninvasive test to identify competent oocytes or embryos. For instance, through the use of RT-qPCR and microarray techniques, numerous studies have correlated changes in CC gene expression with in vitro embryo development and pregnancy outcomes following IVF [5,11]. Nevertheless, while studies in bovine species have identified CC-expressed genes that could potentially serve as markers for oocyte DP [12,13,14], the human CC transcriptome has been found to provide limited predictive value for embryo transfer outcomes [15,16,17]. Thus, no CC transcripts have been identified and validated for clinical application to predict oocyte DP.

Recently, miRNAs have emerged as promising biomarkers for disease diagnosis [18,19] and embryo selection [20,21], given their detectability in circulatory biofluids and culture media. It has been reported that miRNAs are expressed in CCs across several species, including humans [22], cattle [23], mice [24,25], and pigs [26]. Functional analyses indicate that some CC-expressed miRNAs play roles in regulating cumulus expansion [24,26,27], CC apoptosis [22,28], or both [25,29,30]. This suggests that CC-expressed miRNAs may serve as markers for selecting oocytes with high DP. However, only a few studies have demonstrated that CC-expressed miRNAs might regulate oocyte maturation indirectly by modulating CC proliferation and/or steroidogenesis [22,31,32] and by regulating CC apoptosis and cumulus expansion [25]. Studies directly correlating miRNA expression in CCs with oocyte DP remain scarce. The sole study we identified concluded that miRNA expression in bovine CCs cannot be used as an oocyte quality marker [33].

Despite recent advancements identifying new groups of CC-expressed miRNAs [25] and genes [34] that regulate cumulus expansion and/or CC apoptosis and may serve as biomarkers for selecting competent oocytes/embryos, further research is urgently needed to identify and validate CC-expressed genes and miRNAs whose expression levels are directly correlated with oocyte DP. In this study, by conducting mRNA/miRNA sequencing and functional analysis using CCs from mouse cumulus-oocyte-complexes (COCs) with significantly different DP after in vitro or in vivo maturation, we identified and validated novel sets of CC-expressed genes and miRNAs directly correlated with oocyte DP. These newly identified genes/miRNAs can not only serve as markers for selecting competent oocytes/embryos but also contribute to elucidating the mechanisms underlying oocyte maturation. The COC models were adopted because intact COCs best demonstrate the effects of CCs on oocyte DP during IVM, as it has been established that CCs are essential for oocyte cytoplasmic maturation during IVM [35].

## 2. Materials and Methods

All the experiments in this study were carried out in accordance with the relevant guidelines and regulations. Mouse care and handling were performed strictly following the guidelines issued by the Animal Care and Use Committee of the Shandong Agricultural University, P.R. China (permit no. SDAUA-2019-004). All the chemicals and reagents used in this study were obtained from Sigma-Aldrich Corp., St. Louis, MI, USA, unless pointed out otherwise.

### 2.1. Experimental Design

**Figure 1:**
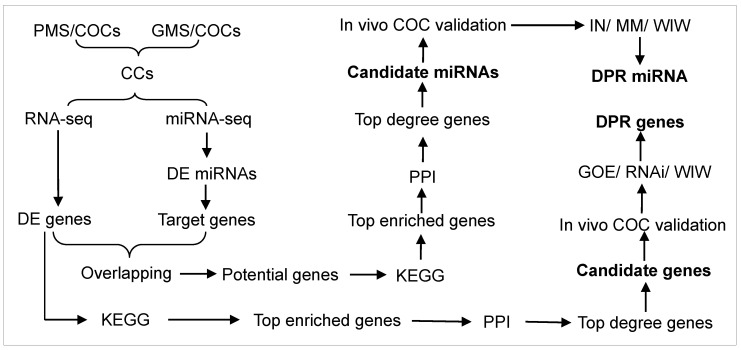
The overall design of the study using a mouse oocyte model. Please refer to the experimental design part of the Materials and Methods section for detailed explanations. Abbreviations: CCs, cumulus cells; COCs, cumulus–oocyte complexes; DE, differentially expressed; DPR, developmental potential-related; GMS, good maturation system; IN, inhibitor; KEGG, Kyoto Encyclopedia of Genes and Genomes; MM, mimic; GOE, gene overexpression; PMS, poor maturation system; PPI, protein–protein interaction; RNAi, RNA interference; WIW, well-in-well activation/embryo culture system that allows tracking the COC origin of CCs and embryos.

Mouse COCs were cultured for 18 h in a good (GMS) or poor maturation system (PMS) before harvesting CCs for isolation of mRNAs and miRNAs (Figure 1). The constituents of maturation media used for PMS and GMS are presented below in Section 2.3. The isolated mRNAs and miRNAs were subjected to RNA- and miRNA-seq to identify differentially expressed (DE) genes and miRNAs, respectively. To select genes, the DE genes were subjected to Kyoto Encyclopedia of Genes and Genomes (KEGG) pathway enrichment, and genes associated with the top enriched pathways (top enriched genes) were subjected to protein–protein interaction (PPI) analysis to obtain the top degree genes. Candidate genes were selected from the top degree genes. To select miRNAs, target genes were predicted from the DE miRNAs and were overlapped with the DE genes identified by RNA-seq to determine the potential genes. The potential genes were subjected to KEGG to obtain the top enriched genes, which were subjected to PPI to get the top genes based on degree score (top degree genes). Candidate miRNAs were predicted from the top degree genes. The candidate genes and miRNAs were subjected to an in vivo COC validation before functional analysis. The in vivo COCs were recovered from mice injected with zearalenone or control mice injected with vehicle DMSO (Refer to Section 2.2 for detailed procedures). For functional analysis, COCs were transfected with siRNA or gene overexpression (GOE) vectors of the candidate genes, or with mimics (MM) or inhibitors (IN) of the candidate miRNAs, and the transfected COCs were cultured in GMS before assessment of post-maturation DP. The genes and miRNAs obtained in the transfection experiment were further validated using the well-in-well (WIW) activation/embryo culture system that allows tracking the COC origin of CCs and embryos to determine the DP-related (DPR) genes or miRNAs.

### 2.2. Oocyte Collection

To collect oocytes for the in vitro model, female mice, 8–10 weeks old, were injected intraperitoneally with 10 IU eCG. Forty-eight hours later, the mice were sacrificed, and the immature oocytes were collected. The COCs that had three or more layers of CCs, and an oocyte with a diameter greater than 70 µm and uniform cytoplasm were selected for the experiment.

To recover oocytes for the in vivo model, female mice, 8–10 weeks old, were injected with zearalenone (ZEN) (1 mg/kg body weight) daily for 5 days. Control mice were injected with a saline solution containing DMSO. At the last injection of ZEN, the mice were injected with 10 IU eCG. After 48 h of eCG injection, the mice were injected with 10 IU hCG. At 13 h after hCG injection, the mice were sacrificed and their mature COCs were collected. This protocol of ZEN treatment was used because our previous study had shown that it significantly impaired DP of mouse oocytes [36].

### 2.3. Oocyte In Vitro Maturation

The COCs were cultured in either PMS or GMS medium for 18 h or 24 h in microdroplets (20–30 COCs per 100 µL droplet) at 37.5 °C in air containing 5% CO_2_. The PMS medium was a simplified MEM (s-MEM), and the GMS medium was composed of s-MEM supplemented with 0.05 IU/mL FSH and 5% fetal bovine serum (FBS, Gibco, Grand Island, NY, USA). The s-MEM contains inorganic salts (1.8 mM CaCl_2_, 0.81 mM MgSO_4_, 5.3 mM KCl, 26.2 mM NaHCO_3_, 117.2 mM NaCl, 1.0 mM NaH_2_PO_4_), 2 mM glutamine, 5.56 mM glucose, 1 mM sodium pyruvate, 4 mg/mL bovine serum albumin, 0.03 mM phenol red, 50 IU/mL penicillin, and 50 µg/mL streptomycin.

### 2.4. Oocyte Activation and Embryo Culture

After maturation for 24 h, the COCs were denuded of CCs in M2 medium containing 0.1% hyaluronidase to obtain cumulus-denuded oocytes (DOs). Then, the DOs were washed 3 times with the activation medium (glucose- and CaCl_2_-free CZB medium containing 10 mM SrCl_2_ and 5 µg/mL cytochalasin B) and cultured for 6 h in 100 µL of the activation medium covered with paraffin oil at 37.5 °C in humidified air with 5% CO_2_. The activation rate was observed under a microscope immediately following activation treatment. When the oocytes showed one pronucleus with one polar body or two pronuclei, they were considered activated. The activated oocytes were selected and washed first with M2, and then with glucose-free CZB medium. Then, the oocytes were cultured in glucose-free CZB. At 24 h of culture, 2-cell rates were observed. At 48 h of culture, the 4-cell rates were examined, and the embryos were cultured in CZB containing 5.6 mM glucose. The morula and blastocyst rates were observed after 24 h and 48 h of culture in glucose-containing CZB medium, respectively.

### 2.5. Glutathione Measurement

This experiment used a GSH and GSSG detection kit (Beyotime, S0053, Beyotime Institute of Biotechnology, Shanghai, China) to detect the glutathione level in oocytes. Forty DOs were placed in a cryopreservation tube containing 30 µL protein removal reagent and subjected to 3 or more repeated freeze-thaw cycles in liquid nitrogen and hot water bath. After 5 min at 4 °C, the samples were centrifuged at 10,000× *g* for 10 min at 4 °C. Then, 20 µL of the supernatant was evenly divided into two parts for immediate measurement of total glutathione (GSX) and oxidized glutathione (GSSG) or stored at −80 °C before measurement. The detection was carried out according to the instructions in the kit, and the content of GSH and GSSG (pmol/oocyte) and their ratio were calculated for each oocyte.

### 2.6. RT-qPCR Measurement of mRNA

Total RNA was extracted from CCs isolated from 50 to 60 COCs using the RNAqueous Micro Total RNA Isolation Kit (AM1931, Ambion, Austin, TX, USA). The isolated RNA was re-suspended in dH_2_O. Reverse transcription was performed using PrimeScript™ RT reagent Kit with gDNA Eraser (Takara, RR047A, TakaraBio, Dalian, China) with a total volume of 20 µL. The steps for reverse transcription are as follows: 4 µL RNA, 1 µL gDNA Eraser, 2 µL 5× gDNA Eraser Buffer, and 3 µL DEPC-dH_2_O were mixed in a 0.2 mL centrifuge tube and reacted in a PCR apparatus at 42 °C for 2 min. After the reaction, 4 µL Prime Script Buffer (5×), 1 µL RT Prime mix, 1 µL Prime Script RT Enzyme Mix, and 4 µL DEPC-dH_2_O were added and allowed to react in a PCR apparatus at 37 °C for 15 min, at 85 °C for 5 s before being stored at −20 °C.

The mRNA was quantified using a Mx3005P real-time PCR (Stratagene, Valencia, CA, USA) and gene-specific primers (Table 1). The amplification reaction was conducted using TB Green Premix Ex TaqTM (Takara, RR420A, TakaraBio, Dalian, China) with a reaction volume of 10 µL. The reaction solution consisted of 1 µL cDNA, 5 µL 2× TB Green Premix Ex TaqII, 0.2 µL ROX, 3.4 µL DEPEC-DH_2_O, and 0.2 µL (10 µM) each of the forward and reverse gene-specific primers. The amplification reaction included (1) pre-denaturation: reaction at 95 °C for 30 s; (2) amplification: reaction for 40 cycles at 95 °C for 5 s and 60 °C for 34 s.

The mRNA levels in cumulus-free oocytes were analyzed by one-step RT-qPCR. Briefly, cumulus-free oocytes were first treated with the Cell Amp Direct Prep Kit for reverse transcription (RT)-PCR (Real Time) and Protein Analysis (TaKaRa, 3733, TakaraBio, Dalian, China), and then, real-time qPCR was performed using the One Step TB Green PrimeScript PLUS RT-PCR Kit (TaKaRa, RR096A, TakaraBio, Dalian, China).

By using 10 different CC samples, we tested the stability of the 8 commonly used housekeeping genes and ranked the expression stability of the analyzed genes using GeNorm (https://seqyuan.shinyapps.io/seqyuan_prosper/, accessed on 30 May 2023). The ranking order of expression stability among the analyzed genes was β-actin > Rplp0 > H2a > Ppib > B2m > Hprt > Gapdh > 18S. We thus used β-actin as the internal reference gene to calculate relative expression with the 2^−ΔΔCT^ method.

### 2.7. RT-qPCR Measurement of miRNA

Total RNA was extracted from CCs from 100 COCs using an Ambion mirVana miRNA Isolation Kit (AM1561, Ambion, Austin, TX, USA). The isolated RNA was suspended with dH_2_O. Reverse transcription was performed using the Takara Bio Reverse-Transcription Kit (638313, Takara Bio, Dalian, China). Briefly, a mixture of 5 µL 2× mRQ buffer, 3.75 mL total RNA (0.25–8 µg/mL), and 1.25 mL mRQ enzyme is incubated at 65 °C for 1 h and then heated at 85 °C for 5 min. The cDNA obtained was then diluted with dH_2_O to 50 µL for subsequent qPCR experiments.

Sequences of primers used in RT-qPCR for miRNAs are shown in Table 2. Real-time qPCR was performed on miRNAs using the Takara Bio RT-qPCR kit (RR820A, Takara Bio, Dalian, China). A 10 µL reaction mixture was prepared according to the manufacturer’s instructions, which consisted of 5 µL 2× TB Green Premix Ex TaqII, 0.4 µL forward primer and 0.4 µL reverse primer, 0.2 µL 50× ROX Reference Dye, 1 µL cDNA, and 3 µL water. The reaction was performed in Mx3005P (Stratagene, Valencia, CA, USA) as follows: denaturation at 95 °C for 30 s, followed by 40 cycles at 95 °C for 5 s and 60 °C for 34 s with U6 as an internal reference gene; miRNA relative expression was calculated using 2^−ΔΔCT^.

### 2.8. Measurement of Mitochondrial Membrane Potential (MMP)

An MMP detection (JC-1) kit (Beyotime Biotechnology Research Institute, Haimen, China) was used to detect MMP. Cumulus-free oocytes were washed three times in M2, placed in a drop of working solution (100 µL M2 and 100 µL JC-1 dye), and stained at 37 °C for 25 min. After washing in JC-1 stain buffer three times, we placed the oocytes on a slide, covered with a coverslip, and observed with a confocal microscope (Dragonfly, Andor Technology Co., Ltd., Belfast, UK). The same oocyte was observed by the Cy3 channel for JC-1 polymer at an emission wavelength of 570 nm (red fluorescence) and by the FITC channel for the JC-1 monomer at an emission wavelength of 512 nm (green fluorescence). The photos were quantitatively analyzed using the Image-Pro Plus software (version 6.0) and the ratio of red fluorescence to green fluorescence was calculated. A lower ratio of red to green fluorescence indicated more serious damage to mitochondria.

### 2.9. Measurement for Cumulus Expansion Area

The COCs were photographed under a microscope before maturation (BM) culture and after maturation (AM) culture for 18 h. The BM and AM cumulus expansion areas of an oocyte were measured, and the ratio of AM/BM cumulus expansion areas was calculated using the ImageJ software (version 6.0).

### 2.10. Flow Cytometry Detection for Apoptosis of CCs

Firstly, CCs from 50 to 60 COCs were dispersed in M2 medium containing 0.1% hyaluronidase using a fine pipette, collected in 1.5 mL centrifuge tube, centrifuged at 1000× *g* for 5 min, and the supernatant was discarded. Then, staining was performed using the FITC Annexin V Apoptosis Detection Kit (556547; BD, Franklin Lakes, NJ, USA) as follows: (1) After 200 µL PBS was added, the samples were centrifuged at 1000× *g* for 5 min; (3) After 200 µL 1× Binding Buffer was added, the samples were centrifuged at 1000× *g* for 5 min; (4) 100 µL 1× Binding Buffer was added to resuspend cells; (5) mixed with 5 µL Annexin V-FITC and 5 µL PI and stained in the dark for 15 min; (6) after staining, 400 µL 1× Binding Buffer was added, mixed thoroughly, and put on ice before detection; (7) LSRFortessa (BD, Becton, Dickinson and Company, Franklin Lakes, NJ, USA) was used to analyze the fluorescence values of 10,000 cells.

### 2.11. Illumina HiSeq Sequencing of mRNA and miRNA in CCs

**Total RNA extraction**: We extracted total RNA from CC samples using a TRlzol Reagent kit (Life technologies, Carlsbad, CA, USA) for mRNA and miRNA sequencing. While each CC sample for mRNA sequencing contained CCs from approximately 300 COCs, each sample for miRNA sequencing contained CCs from approximately 900 COCs. We collected approximately 120 COCs on each experimental day, and approximately 30 were used for mRNA and 90 for miRNA extraction. We centrifuged the recovered CCs (2000× *g* for 5 min) and stored them in liquid nitrogen before mRNA/ miRNA extraction.

**Evaluation of RNA integrity**: We measured RNA concentration and purity using NanoDrop 2000 (Thermo Fisher Scientific, Wilmington, NC, USA). We assessed RNA integrity using the RNA Nano 6000 Assay Kit of the Agilent Bioanalyzer 2100 system (Agilent Technologies, Santa Clara, CA, USA).

**Library preparation and sequencing for RNA-seq**: Biomarker Technologies (Beijing, China) carried out the library preparation and sequencing. They used a total amount of 1 µg RNA per sample as input material for the RNA sample preparations. They generated sequencing libraries using the Hieff NGS Ultima Dual-mode mRNA Library Prep Kit for Illumina from Yeasen Biotechnology (Shanghai) Co., Ltd., Shanghai, China. following the manufacturer’s recommendations and added index codes to attribute sequences of each sample. Briefly, they purified mRNA from total RNA using poly-T oligo-attached magnetic beads. They synthesized the first strand of cDNA and then the second strand of cDNA. They converted the remaining overhangs into blunt ends through exonuclease/polymerase activities. To prepare for hybridization, they ligated the NEBNext adaptor with a hairpin loop structure after adenylation of 3′ ends of DNA fragments. They purified library fragments with the AMPure XP system (Beckman Coulter, Beverly, MA, USA). Then, they incubated 3 µL USER Enzyme (NEB, Ipswich, MA, USA) with size-selected, adaptor-ligated cDNA at 37 °C for 15 min followed by 5 min at 95 °C before PCR. They performed PCR with Phusion High-Fidelity DNA polymerase, Universal PCR primers, and Index (X) Primer. Finally, they purified the PCR products with the AMPure XP system and assessed library quality on the Agilent Bioanalyzer 2100 system. They sequenced the libraries on an Illumina NovaSeq platform to generate 150 bp paired-end reads, according to the manufacturer’s instructions.

**Library preparation and sequencing for miRNA-seq**: A VAHTSTM Small RNA Library Prep Kit for Illumina (NR801-02, Nanjing Vazyme Biotech Co., Ltd., Nanjing, China) was used to generate sequencing libraries following the manufacturer’s instructions. In brief, 3′ and 5′ adapters were first ligated, followed by reverse transcription and PCR amplification. VAHTSTM DNA Clean Beads (N411-03, Nanjing Vazyme Biotech Co., Ltd.) were used to purify the PCR products according to the manufacturer’s instructions, and the PCR-purified products were subjected to PAGE gel electrophoresis, and the gel was cut to recover the small RNA library. The library was quality-checked by the Qsep-400 method. The library was sequenced on the Illumina NovaSeq6000 platform to generate single-end sequences according to the manufacturer’s instructions.

### 2.12. Bioinformatic Analysis

**Quality control**: Firstly, we processed raw data (raw reads) of fastq format through in-house perl scripts. During this step, clean data (clean reads) were obtained after removing reads containing adapter, reads containing ploy-N, and low-quality reads from raw data. At the same time, Q20, Q30, GC-content, and the sequence duplication level of the clean data were calculated. All the downstream analyses were performed based on clean data with high quality.

**Analysis of differential expression**: We used the DESeq2 to perform the differential expression analysis between the two conditions/groups. DESeq2 provided statistical routines for determining differential expression in digital gene expression data using a model based on the negative binomial distribution. We adjusted the resulting *p*-values using the Benjamini and Hochberg’s approach for controlling the false discovery rate. Genes with an adjusted *p*-value < 0.05 and Fold Change > 2 and miRNAs that had values of FC > 1.5 and adjusted *p*-value < 0.05 revealed by DESeq2 were considered as differentially expressed.

**Cluster analysis of DE mRNAs and miRNAs**: We used the fragments per kilobase per million reads (FPKM) and transcripts per million (TPM) values to represent the level of mRNA and miRNA expression, respectively, in CCs from different treatments to carry out the hierarchical clustering analysis. The normalization of miRNA expression levels between PMS and GMS was carried out using the normalization formula: microRNA read count × 10^6^/total reads. We conducted the normalization of mRNA expression using the normalization formula: Total exon fragments/mapped reads (millions) × exon length (KB). The different cluster grouping information was expressed with different colors. The heat maps of DE mRNAs and miRNAs were drawn using the MeV 4.9.0 software.

**Analyses of KEGG pathway enrichment and PPI**: We conducted the KEGG (http://en.wikipedia.org/wiki/KEGG, accessed on 9 August 2023) and PPI (String Database, http://string-db.org/, accessed on 9 August 2023) analyses on the candidate genes. We used the software of DAVID Bioinformatics Resources 6.8 for KEGG analysis, and *p* < 0.05 was considered significantly enriched. To generate the PPI network, we selected the ‘none/query proteins only’ option in the 1st shell and selected the ‘none’ option in the 2nd shell. We considered only PPIs with a medium confidence score of 0.4, and we used the software Cytoscape v3.6.1 (https://www.cytoscape.org/, accessed on 9 August 2023) to visualize the protein interaction network. We identified the hub proteins based on the node degree that represents the number of interactions (edges) the node has, and we selected only proteins with a node degree of 40 as top degree genes.

### 2.13. Transfection of COCs with siRNA or Plasmids

The siRNA and its negative control were synthesized by Guangzhou RiboBio Biotech Co., Ltd., Guangzhou, China. The siRNA and negative control were diluted to 20 µM in RNase-free water, aliquoted, and stored at −20 °C prior to use. The transfection medium was prepared as follows: (1) 2.5 µL of siRNA was added to 22.5 µL of Opti-MEM^®^ I Reduced Serum Medium (5198-5042, Life Technologies, Gaithersburg, MD, USA) and incubated in the dark for 5 min; (2) 1.5 µL of Lipofectamine^®^ RNAiMAX Reagent (137-78500, Invitrogen, Carlsbad, CA, USA) was added to 23.5 µL of Opti-MEM and incubated in the dark for 5 min; (3) 12.5 µL from each of (1) and (2) were mixed to form a 25 µL mixture and incubated in the dark for 5 min; (4) 25 µL of the mixture from step (3) was added to 225 µL of GMS culture medium to form a 250 µL transfection solution, with the final transfection concentration of 100 nM. We added 100 µL of the transfection solution to each culture well with 20–30 COCs, and we collected the oocytes after transfection for 24 h. The silencing efficiency of siRNA sequences was confirmed first in preliminary experiments by RT-qPCR analysis of mRNA levels in CCs following transfection, and we used only those siRNA sequences that significantly downregulated the mRNA level compared with that in negative controls.

Plasmid DNA and its negative control were synthesized in Shandong Jinbaiao Biotechnology Co., Ltd., Jinan, China. Plasmid DNA and its negative control were stored at 500 ng/µL at −20 °C. The transfection medium was prepared as follows: (1) 4.8 µL Opti-MEM^®^ I Reduced Serum Medium (51985042, Life technologies, Gaithersburg, MD, USA) and 0.2 µL Lipofectamine™ 3000 (L3000008, Invitrogen, Carlsbad, CA, USA) were mixed well. (2) Mix 4.4 µL Opti-MEM, 0.4 µL plasmid DNA, and 0.2 µL P3000™ (L3000008, Invitrogen, Carlsbad, CA, USA) were mixed. Mixtures (1) and (2) were mixed well and allowed to set for 15 min. (3) A total of 90 µL GMS culture medium was added into the mixture and mixed to form 100 µL transfer solution, and the final concentration of plasmid DNA was 2 ng/µL. Then, 20–30 COCs were placed in each culture well containing 100 µL transfer solution. At 6 h of transfection culture, COCs were washed in M2 and GMS medium and cultured in GMS medium for 18 h.

### 2.14. Transfection of COCs with miRNA Mimics

Mimic and its negative control (MC) were synthesized by Guangzhou Ribo Biotech Co., Ltd., Guangzhou, China. After being centrifuged briefly, RNase-free water was added to diluted Mimic and MC to a 20 μM storage solution. Mimic and MC were then divided into aliquots and stored at −20 °C. The transfection medium was prepared as follows: (1) 1.25 µL of Mimic were added to 23.75 µL of Opti-MEM^®^ I Reduced Serum Medium and incubated in the dark for 5 min; (2) 1.5 µL of Lipofectamine^®^ RNAiMAX Reagent (Life technologies, Carlsbad, CA, USA) were added to 23.5 µL of Opti-MEM and incubated in the dark for 5 min; (3) 12.5 µL from each of the mixtures (1) and (2) were mixed to form a 25 µL mixture, which was incubated in the dark for 5 min; (4) 225 µL of GMS culture medium were added to the incubated 25 µL mixture to form a 250 µL transfection solution, with a final transfection concentration of 50 nM. Finally, 100 µL of the transfection solution was added to each culture well, together with 20–30 COCs, and incubated for transfection for 24 h.

### 2.15. Activation and Embryo Culture in the WIW System

A 48-well culture plate was used to prepare the WIWs. Sixteen WIWs, which were approximately 0.5 mm width and 0.3 mm depth, were created in each well of the 48-well plate. Methods for making the WIWs were previously reported by this lab [37]. Each of the wells with 16 WIWs was filled with 200 µL of activation or embryo culture medium and covered with mineral oil. Following normal (group) maturation culture in PMS or GMS for 24 h, the COCs were denuded of CCs individually, and the CCs collected from different COCs were stored separately in different test tubes. For activation, 16 DOs were treated individually in the 16 WIWs, and then, the activation-treated DOs were cultured individually in 16 WIWs containing embryo culture medium for embryo development. At the end of the embryo culture, CCs from COCs that developed to 1- or 2-cell (≤2C) embryos and the CCs from COCs that developed to 4-cell or beyond (≥4C) embryos were pooled separately for RT-PCR analysis of gene/miRNA levels.

### 2.16. Data Analysis

Unless pointed out specifically, we repeated each treatment three times. We arc sine transformed the percentage data before analyzing them using an independent-sample Student’s *t*-test. We used the Statistics Package for Social Science (SPSS 20, SPSS, Inc., Chicago, IL, USA) to conduct the data analysis. We expressed the data as mean ± SEM and we considered a difference significant when the *p*-value was < 0.05.

## 3. Results

### 3.1. The Establishment of In Vitro CC Models Using In Vitro Matured COCs with Significantly Different DP

**Figure 2:**
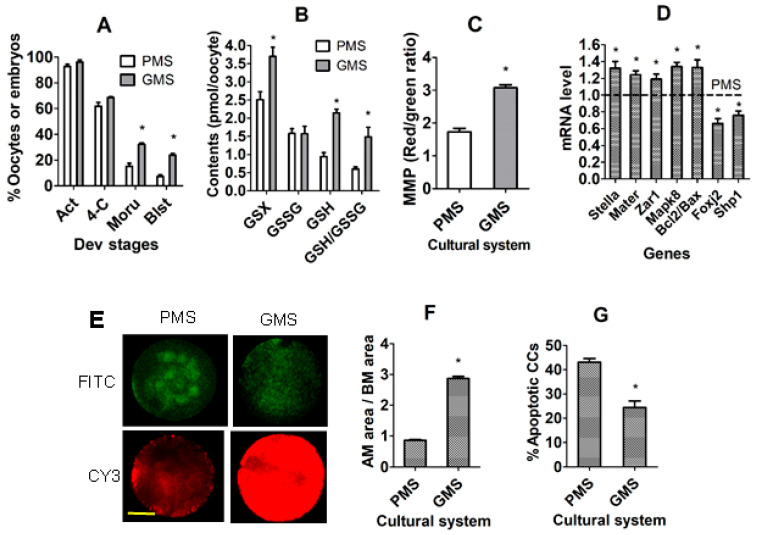
Oocyte quality and developmental potential after mouse COCs were matured with poor (PMS) or good maturation system (GMS). Graph (**A**) shows percentages of activated oocytes (Act), and 4-cell (4-C), morula (Moru), and blastocyst (Blst) parthenogenetic embryos, calculated from total oocytes cultured for maturation. Each treatment was repeated 4 times, with each replicate including 20–30 oocytes. Graph (**B**) shows contents (pmoL/oocyte) of total glutathione (GSX), oxidized glutathione (GSSG), reduced glutathione (GSH), and the ratio of GSH/GSSG. Each treatment was repeated 4 times with each replicate containing 40 oocytes. Graph (**C**) shows mitochondrial membrane potential (MMP, red/green ratio). Each treatment was repeated 3 times, with each replicate including 20–30 oocytes. Graph (**D**) shows mRNA levels of developmental potential- and apoptosis-related genes. Each treatment was repeated 3–4 times, and each replicate contained 30 oocytes. Panel (**E**) shows confocal images showing JC-1 staining intensity in oocytes matured with PMS or GMS. The upper and lower images are for the same oocytes observed in CY3 (red fluorescence) and FITC (green) channels, respectively. Bar is 20 µm. Graph (**F**) shows the ratio of after maturation (AM)/before maturation (BM) area of cumulus expansion after COCs were matured with PMS or GMS. Each treatment was repeated 4 times, and each replicate contained 20–30 oocytes. Graph (**G**) shows flow cytometry-measured percentages of apoptotic (including both early and late apoptotic) CCs after COCs were matured with PMS or GMS. Each treatment was repeated 4 times, and each replicate contained 50–60 oocytes. * Indicates a significant difference (*p* < 0.05) between PMS and GMS groups.

Mouse COCs were matured in PMS or GMS for 24 h before Sr^2+^ activation for embryo development or matured for 18 h before examination for oocyte quality parameters. Both percentages of parthenogenetic morulae and blastocysts were significantly higher in oocytes matured with GMS than with PMS (Figure 2A). Although the level of oxidized GSH (GSSG) did not differ, levels of total GSH, reduced GSH and GSH/GSSG ratio were significantly higher in GMS oocytes than in PMS oocytes (Figure 2B). The mitochondrial membrane potential (MMP) was significantly higher in GMS oocytes than in PMS oocytes (Figure 2C,E). While the mRNA level of DP-beneficial genes was higher, that of the DP-detrimental genes was significantly lower in GMS oocytes than in PMS oocytes (Figure 2D). Furthermore, the area of cumulus expansion was larger (Figure 2F), and the percentage of apoptotic CCs were lower (Figure 2G) significantly in COCs after maturation in GMS than in PMS. Thus, the results demonstrate that the PMS culture impaired DP while causing oxidative stress in oocytes, suggesting that CCs from the COCs matured with GMS and PMS can be used as CC models to identify DE genes and miRNAs that regulate oocyte DP.

### 3.2. Identification of DE Genes in CCs Between PMS- and GMS-Matured Oocytes

**Figure 3:**
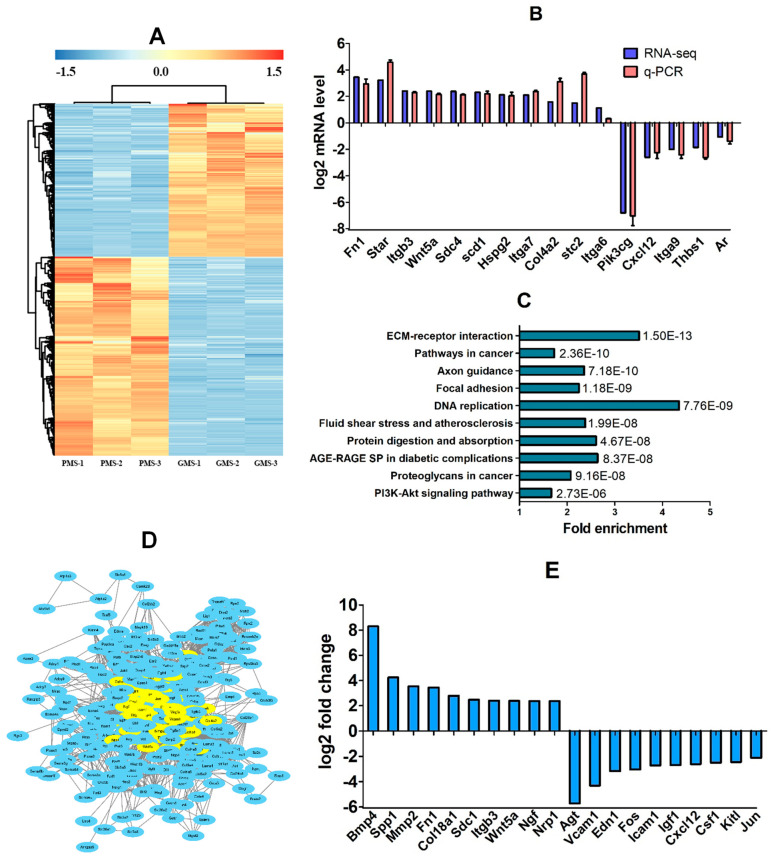
Identification and bioinformatic analysis of differentially expressed (DE) genes in CCs between oocytes matured with PMS and GMS. Graph (**A**) shows a heat map showing the hierarchical clusters of DE genes identified by RNA-seq. Three samples from the PMS group (PMS-1, -2, and -3) and three from the GMS group (GMS-1, -2, and -3) were analyzed. Each CC sample for RNA-seq contained CCs from approximately 300 COCs. While blue color indicates relatively upregulated, red color indicates downregulated genes. Only genes with a significant change of *p* < 0.05 and fold change (FC) > 2 are shown. Graph (**B**) shows RT-qPCR verification of mRNA-seq results for selected genes. The RT-qPCR was performed using the PMS and GMS samples obtained in exactly the same way as samples used for RNA-seq. Each treatment was repeated three times, with each replicate containing CCs from 50 to 60 oocytes. Levels of all the mRNAs differed significantly (*p* < 0.05) between PMS and GMS. Graph (**C**) shows the top 10 KEGG pathways significantly (*p* < 0.05) enriched by the 3094 DE genes. The 10 top KEGG pathways contained 320 genes. Bars are the fold enrichment of pathways, and the values next to the bars are the *p*-values. SP: Signaling pathway. Graph (**D**) shows a PPI network constructed with STRING (https://string-db.org/) using proteins encoded by the 320 candidate genes; the network contains the 55 proteins with a degree of ≥40 (in yellow). Graph (**E**) shows the 20 top degree genes, selected from the 55 PPI-selected genes with 10 up- and 10 downregulated.

RNA-seq was performed to identify DE genes expressed in CCs between PMS- and GMS-matured oocytes. The RNA-seq identified a total of 3094 DE genes (*p* < 0.05, FC > 2), with 1348 upregulated and 1746 downregulated in GMS oocytes relative to PMS oocytes (Figure 3A). To validate the RNA-seq results, RT-qPCR was carried out on selected mRNAs. The results revealed the same expression pattern as that detected by RNA-seq in all the 16 genes examined (Figure 3B), suggesting that our RT-qPCR results were in good agreement with our RNA-seq results and further confirming the significant differences in gene expression between GMS and PMS CCs.

### 3.3. KEGG and PPI Analyses on DE Genes

Firstly, the DE genes identified by RNA-seq were subjected to KEGG enrichment analysis. The 3094 DE genes were significantly enriched in 89 KEGG pathways, from which the top 10 significant pathways were selected in the *p*-value order (Figure 3C). The 10 top significant pathways contained 320 genes. We then subjected the 320 genes to a PPI analysis. The results selected 55 genes with a degree ≥ 40 (Figure 3D), from which 20 candidate genes (10 up- and 10 down-regulated) were selected according to the FC order (Figure 3E).

### 3.4. The Establishment of In Vivo CC Models Using In Vivo Matured COCs with Significantly Different DP

**Figure 4:**
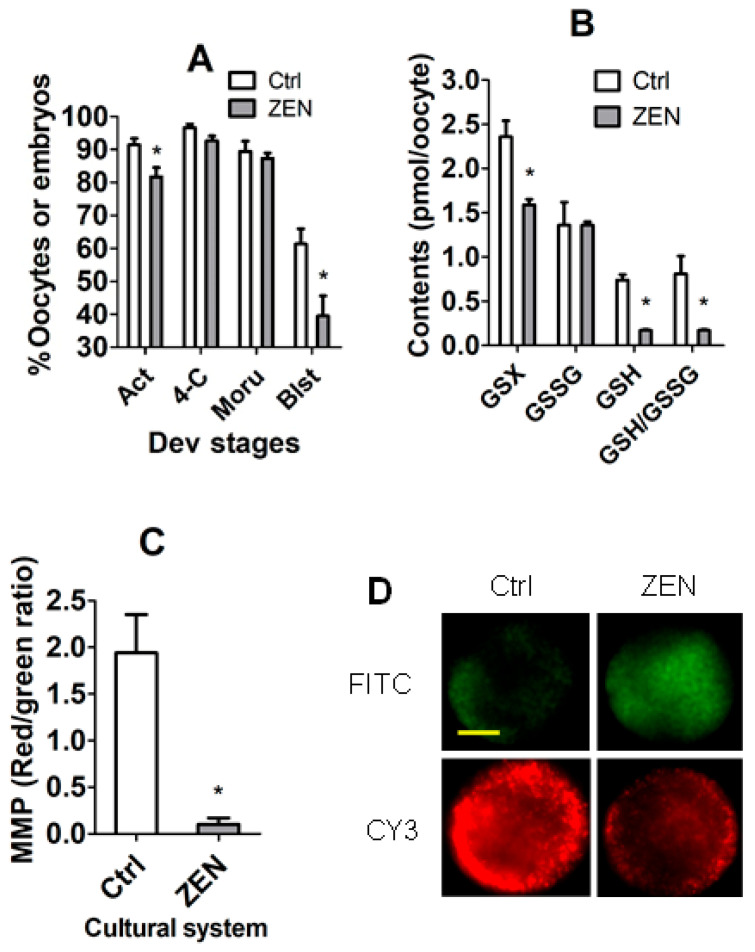
Establishment of the in vivo COC models with significantly different oocyte developmental potential. Female mice were injected with zearalenone (ZEN) and control (Ctrl) mice were injected with vehicle DMSO before superovulation to recover in vivo matured COCs. The COCs obtained were either activated for embryo development or measured for glutathione contents or mitochondrial membrane potential (MMP). Graph (**A**) shows percentages of activated (Act) oocytes, 4-cell (4-C), morula (Moru), and blastocyst (Blst) parthenogenetic embryos after oocytes from ZEN and Ctrl mice were Sr^2+^-activated. Each treatment was repeated eight times and each replicate contained 20–30 oocytes. The percentages of Act, 4-C, Moru and Blst were all calculated from oocytes treated for activation. Graph (**B**) shows intra-oocyte contents of total glutathione (GSX), oxidized glutathione (GSSG), reduced glutathione (GSH), and ratio of GSH/GSSG in Ctrl and ZEN-treated mice. Each treatment was repeated three times with each replicate containing 40 DOs. Graph (**C**) shows MMP. Each treatment was repeated three times with each replicate including 30–50 oocytes. Panel (**D**) shows confocal images showing JC-1 staining intensity in oocytes from ZEN or Ctrl mice. The upper and lower images are for the same oocytes observed in CY3 (red fluorescence) and FITC (green) channels, respectively. Bar is 24 µm. * Indicates a significant difference (*p* < 0.05) between Ctrl and ZEN groups.

To establish the in vivo COC models, mature COCs were collected from mice injected with either ZEN or vehicle (control) before examination for oocyte DP, intra-oocyte glutathione concentration, and mitochondrial membrane potential (MMP). After SrCl_2_ activation, rates of activation and parthenogenetic blastocysts were significantly lower in oocytes from ZEN-treated mice than in oocytes from control mice, although rates of 4-cell and morula parthenogenetic embryos did not differ significantly (Figure 4A). Levels of total and reduced glutathione and the ratio of reduced/oxidized glutathione were significantly lower in ZEN oocytes than in control oocytes (Figure 4B). Furthermore, the level of MMP decreased dramatically to almost none following ZEN treatment of mice (Figure 4C,D). Thus, the results demonstrate that ZEN treatment of female mice significantly impaired DP while causing oxidative stress in oocytes, suggesting that CCs from mature COCs from ZEN-treated and control mice can be used as CC models to validate candidate genes and miRNAs that regulate oocyte DP.

### 3.5. Validation of Candidate Genes Using In Vivo COCs and by RNAi or Gene Overexpression (GOE)

**Figure 5:**
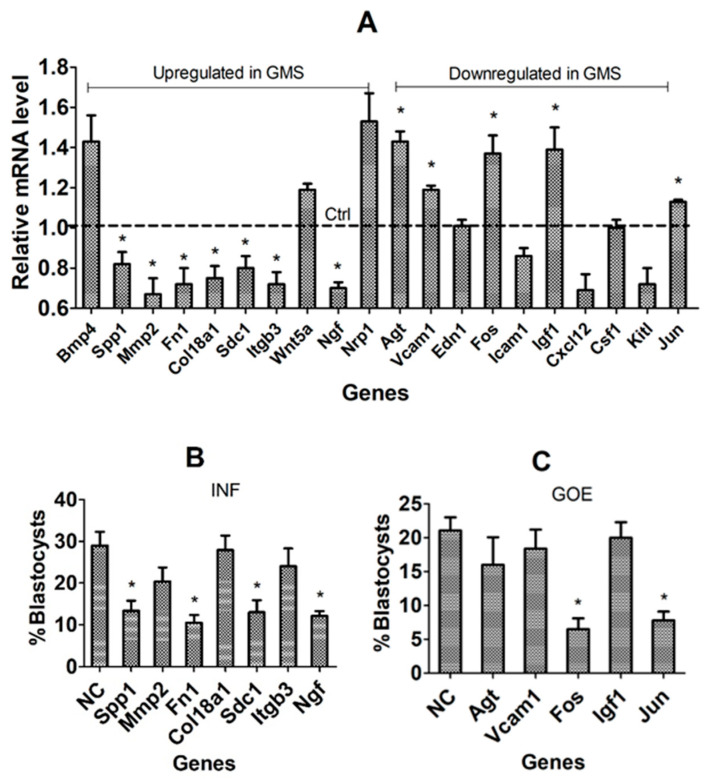
Validation of candidate genes using in vivo COCs and by RNAi (INF) or gene overexpression (GOE). Graph (**A**) shows RT-qPCR-revealed mRNA levels of up- or down-regulated candidate genes in CCs from in vivo matured COCs obtained from ZEN-treated and control (Ctrl) mice. Each treatment was repeated 3–6 times with each replicate including CCs from 50–60 COCs. The mRNA level in Ctrl mice was set to one (dotted line), and that of the ZEN-treated mice was expressed relative to one. Graphs (**B**,**C**) show percentages of parthenogenetic blastocysts after COCs were transfected with siRNAs and GOE plasmids, respectively, of different genes during in vitro maturation with GMS. Each treatment was repeated five times with each replicate, including 20–30 COCs. The percentages of blastocysts were calculated from oocytes transfected with siRNA or GOE plasmids. * Indicates a significant difference (*p* < 0.05) from the Ctrl or NC groups.

Expression of the 20 candidate genes selected in the above experiments was compared between the ZEN-treated and control CCs to validate them as the DPR genes. Whereas the upregulated genes in our COCs matured with GMS should be DP-beneficial genes, the downregulated genes in these COCs should be DP-detrimental genes. Among the 10 would-be DP-beneficial genes, 7, including Spp1, Mmp2, Fn1, Col18a1, Sdc1, Itgb3, and Ngf, were downregulated significantly in the ZEN-treated COCs (Figure 5A), confirming that they are DP-beneficial. Among the 10 would-be DP-detrimental genes, 5, including Agt, Vcam1, Fos, Igf1 and Jun, were upregulated significantly in the ZEN-treated COCs, confirming that they are DP-detrimental. Together, our experiments using the in vivo CC models validated 12 DPR genes.

When RNAi was performed on the 7 DP-beneficial genes during in vitro maturation of COCs with GSM, silencing 4 genes, including Spp1, Fn1, Sdc1, and Ngf, significantly decreased the percentage of blastocyst formation following Sr^2+^ activation of oocytes (Figure 5B), confirming that these 4 genes are DP-beneficial. When GOE was conducted on the 5 DP-detrimental genes, the overexpression of Fos and Jun significantly decreased parthenogenetic blastocyst rates (Figure 5C), confirming that these two genes are DP-detrimental. Altogether, the results functionally verified 6 DPR genes.

### 3.6. Identification of DE miRNAs in CCs Between PMS- and GMS-Matured Oocytes

**Figure 6:**
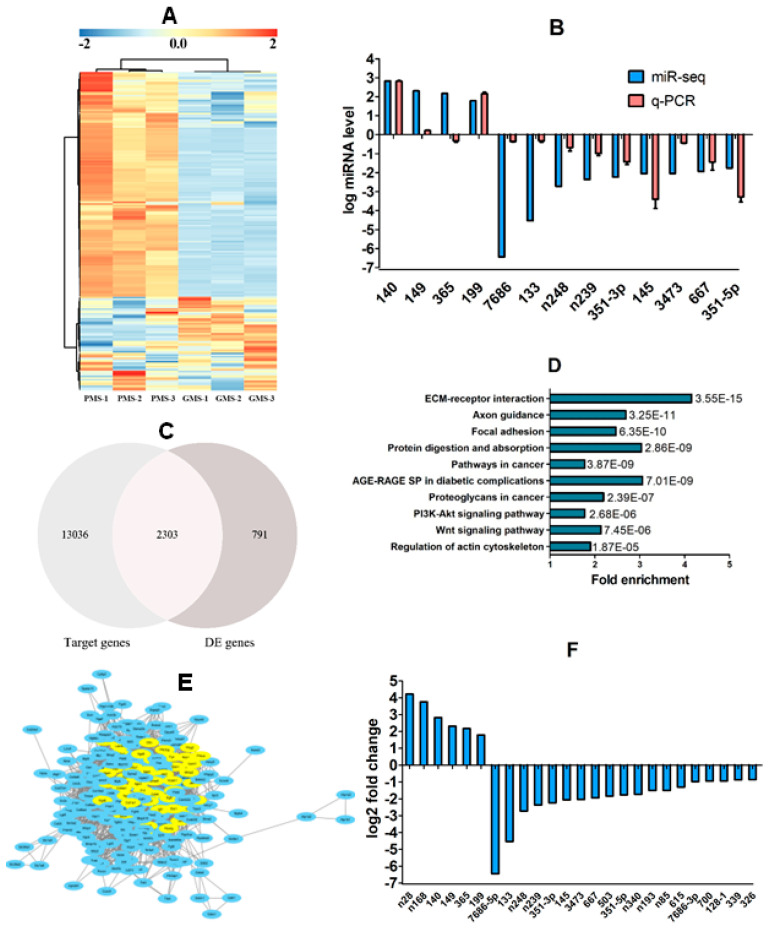
Identification and bioinformatic analysis of differentially expressed (DE) miRNAs in CCs between oocytes matured with PMS and GMS. Graph (**A**) shows a heat map showing the hierarchical clusters of DE miRNAs identified by miRNA-seq. Three samples from the PMS group (PMS-1, -2, and -3) and three from the GMS group (GMS-1, -2, and -3) were analyzed. Each sample for miRNA sequencing contained CCs from approximately 900 COCs. While blue color indicates relatively upregulated, red color indicates downregulated miRNAs. Only miRNAs with a significant change of *p* < 0.05 and fold change (FC) > 1.5 are shown. Graph (**B**) shows RT-qPCR verification of miRNA-seq results for selected miRNAs. The RT-qPCR was performed using the PMS and GMS; the samples were obtained exactly in the same way as the samples used for miRNA-seq. Each treatment was repeated 3–5 times, with each replicate containing CCs from 100 oocytes. Levels of all the miRNAs differed significantly (*p* < 0.05) between PMS and GMS. Graph (**C**) shows that an overlap between the 3094 DE genes and the 15,339 genes targeted by the 194 DE miRNAs selected 2303 candidate genes. Graph (**D**) shows the top 10 KEGG pathways significantly (*p* < 0.05) enriched by the 2303 candidate genes, which contained 275 candidate genes. Bars are the fold enrichment of pathways, and the values next to the bars are the *p*-values. Graph (**E**) shows a PPI network constructed with STRING (https://string-db.org/) using proteins encoded by the 275 candidate genes; the network contains the 51 proteins with a degree of ≥40 (in yellow). Graph (**F**) shows the 25 top FC miRNAs selected from the 63 miRNAs targeted by the 51 PPI-selected genes, with 6 up- and 19 downregulated.

A miRNA-seq was conducted to identify DE miRNAs expressed in CCs between PMS- and GMS-matured oocytes. The miRNA-seq identified a total of 194 DE miRNAs (*p* < 0.05, FC > 1.5) with 94 upregulated and 100 downregulated in GMS oocytes relative to PMS oocytes (Figure 6A). To validate the miRNA-seq results, RT-qPCR was performed on 13 selected miRNAs. The results found the same expression pattern as that detected by miRNA-seq in 12 miRNAs except for miR-365-2-5p (Figure 6B). The results suggest that the RT-qPCR results were in general agreement with our miRNA-seq data and further confirm the significant differences in miRNA expression between the GMS and PMS CCs.

### 3.7. Bioinformatic Analysis on the DE miRNAs

An overlap between the 15,339 genes targeted by the 194 DE miRNAs and the 3094 DE genes identified by RNA-seq produced 2303 candidate genes (Figure 6C). The 2303 candidate genes were significantly enriched in 38 KEGG pathways, from which the top 10 pathways were selected according to the *p*-value, as shown in Figure 6D. The 10 top pathways contained 275 genes. When the 275 genes were subjected to PPI analysis, 51 top-degree genes with a degree of ≥40 were obtained (Figure 6E). The 51 top-degree genes were targeted by 63 miRNAs, from which 10 upregulated and 20 downregulated were selected in the FC order. After 5 miRNAs that showed the same tendency of expression as their target genes and were excluded, 25 candidate miRNAs were selected (Figure 6F). Because miR-365-2-5p showed different expression patterns between RNA-seq and RT-qPCR (Figure 5B), 24 candidate miRNAs (5 upregulated and 19 downregulated) were subjected to further validation. Among the 12 DPR genes we validated using the in vivo CC models, 4 (Mmp2, Fn1, Col18a1, and Itgb3) were found to be targeted by 6 of the 24 candidate miRNAs (miR-615-5p, miR-667-5p, n-miR-248, n-miR-85, miR-351-5p, and miR-7686-5p).

### 3.8. Validation of Candidate miRNAs Using In Vivo COC Models and by miR Mimics

**Figure 7:**
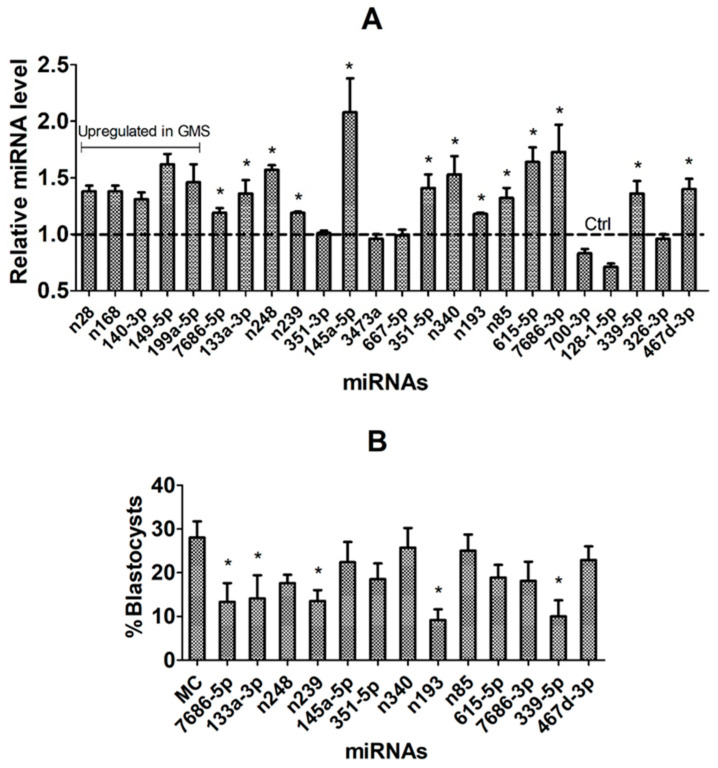
Validation of candidate miRNAs using in vivo COC models and miR mimics. Graph (**A**) shows RT-qPCR-revealed miRNA levels of up- or downregulated candidate miRNAs in CCs from in vivo matured COCs obtained from ZEN-treated and control (Ctrl) mice. Each treatment was repeated 3–6 times with each replicate including CCs from 100 COCs. The miRNA level in Ctrl mice was set to one (dotted line), and that of the ZEN-treated mice was expressed relative to one. Graph (**B**) shows percentages of parthenogenetic blastocysts after COCs were transfected with miRNA mimics or mimic control (MC) of different miRNAs during in vitro maturation with GMS. Each treatment was repeated 7 times, with each replicate including 20–30 COCs. The percentages of blastocysts were calculated from oocytes transfected. * Indicates a significant difference (*p* < 0.05) from the Ctrl or MC groups.

Expression of the 24 candidate miRNAs selected in the above experiments was compared between the ZEN-treated and control CCs to validate them as the DPR miRNAs. Because the above experiments showed that COCs matured with GMS exhibited significantly higher DP than those matured with PMS, we considered the upregulated miRNAs in the COCs matured with GMS as DP-beneficial miRNAs, whereas the downregulated miRNAs in these COCs as DP-detrimental. None of the 5 would-be DP-beneficial miRNAs was downregulated significantly in the ZEN-treated COCs (Figure 7A). Among the 19 would-be DP-detrimental miRNAs, 13, including miR-7686-5p, miR-133a-3p, novel-miR-248, novel-miR-239, miR-145a-5p, miR-351-5p, novel-miR-340, novel-miR-193, novel-miR-85, miR-615-5p, miR-7686-3p, miR-339-5p and miR-467d-3p, were significantly upregulated in the ZEN-treated COCs, suggesting that they are DP-detrimental. Together, our experiments using the in vivo CC models validated 13 DP-detrimental miRNAs.

When mimics of the 13 DP-detrimental miRNAs were transfected during in vitro maturation of COCs with GSM, transfection of mimics of 5 miRNAs, including miR-7686-5p, miR-133a-3p, novel-miR-239, novel-miR-193 and miR-339-5p, significantly decreased the percentage of blastocyst formation following Sr^2+^ activation of oocytes (Figure 7B), confirming that these 5 miRNAs are oocyte DP-detrimental. Together, this study identified and validated five oocyte DP-detrimental miRNAs.

### 3.9. Further Validation of Candidate Genes/miRNAs Using the WIW System of Activation and Embryo Culture

**Figure 8:**
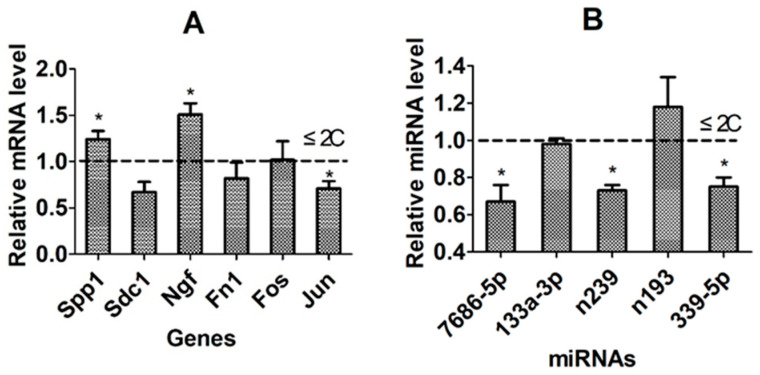
Further validation of candidate genes/miRNAs using the WIW system of activation and embryo culture. Graphs (**A**,**B**) compare mRNA and miRNA levels, respectively, of different genes/miRNAs in CCs between COCs that developed to ≥4-cell embryos and COCs that developed to ≤2-cell (2C) embryos following the WIW activation and embryo culture. The value of ≤2C COCs was set to 1 (dotted line), and that of the ≥4-cell embryo COCs was expressed relative to 1. Each treatment was repeated 3–8 times, with each replicate containing 50–60 COCs. * Indicates a significant difference (*p* < 0.05) between COC groups.

The genes/miRNAs validated from the above experiments were further validated using the WIW activation/embryo culture system that allows identification of the COC origin of CCs and embryos. Among the six DP-related genes validated in the above experiments, while the mRNA levels of DP-beneficial Spp1 and Ngf were significantly higher, that of the DP-detrimental Jun was significantly lower in CCs from the GMS-matured COCs that developed to ≥4C embryos than in CCs from COCs that developed to ≤2C embryos (Figure 8A). Among the 5 DP-detrimental miRNAs validated in the above experiments, levels of miR-7686-5p, novel-miR-239, and miR-339-5p were significantly lower in CCs from the GMS-matured COCs that developed to ≥4C embryos than in CCs from COCs that developed to ≤2C embryos (Figure 8B). Together, the results validated Spp1 and Ngf as DP-beneficial while Jun as a DP-detrimental gene, and miR-7686-5p, novel-miR-239, and miR-339-5p as DP-detrimental miRNAs.

## 4. Discussion

The present study demonstrated that mouse COCs matured using the PMS and GMS systems exhibited significant differences in DP. Specifically, maturation with PMS significantly impaired DP while inducing oxidative stress (as evidenced by a decreased GSH/GSSG ratio and reduced MMP level) in oocytes compared to maturation with GMS. These findings suggest that CCs from COCs matured with GMS and PMS can serve as in vitro models for identifying DE genes and miRNAs regulating oocyte DP. Furthermore, the results of this study also revealed that treatment of female mice with ZEN significantly impaired oocyte DP while causing oxidative stress compared to untreated control mice. This indicates that CCs from mature COCs derived from ZEN-treated and control mice can function as in vivo models for validating candidate genes and miRNAs regulating oocyte DP.

Among the seven miRNAs identified in mouse CCs in our previous study [25], miR-149-5p, and miR-31-5p have been validated to promote cumulus expansion while inhibiting CC apoptosis in pig oocytes [38]. miR-145-5p was found to be detrimental to cumulus expansion and CC viability in mice in our prior research [25], and its downregulation by GDNF in CCs enhanced human oocyte maturation and CC viability [30]. RNA-seq analysis conducted in our earlier study [34] identified Vegfa and Cdh5 as beneficial genes for cumulus expansion and CC viability, whereas Vcam1 and Ctgf were identified as detrimental genes. Park et al. [39] observed that IVM of pig COCs using an agarose matrix resulted in significantly higher blastocyst formation, cumulus expansion, and Vegfa gene expression. Xu et al. [40] reported that the Cdh5 gene was significantly upregulated in mouse CCs when oocytes developed to the blastocyst stage. The expression of the VCAM1 gene was found to increase significantly with aging and apoptosis during long-term culture of human CCs [41]. Additionally, the CTGF gene was significantly upregulated in CCs associated with low-quality human oocytes compared to those associated with high-quality oocytes [42]. Collectively, these data suggest that genes identified using mouse CCs as potential markers for oocyte quality can be validated in other mammals, including humans.

**Figure 9:**
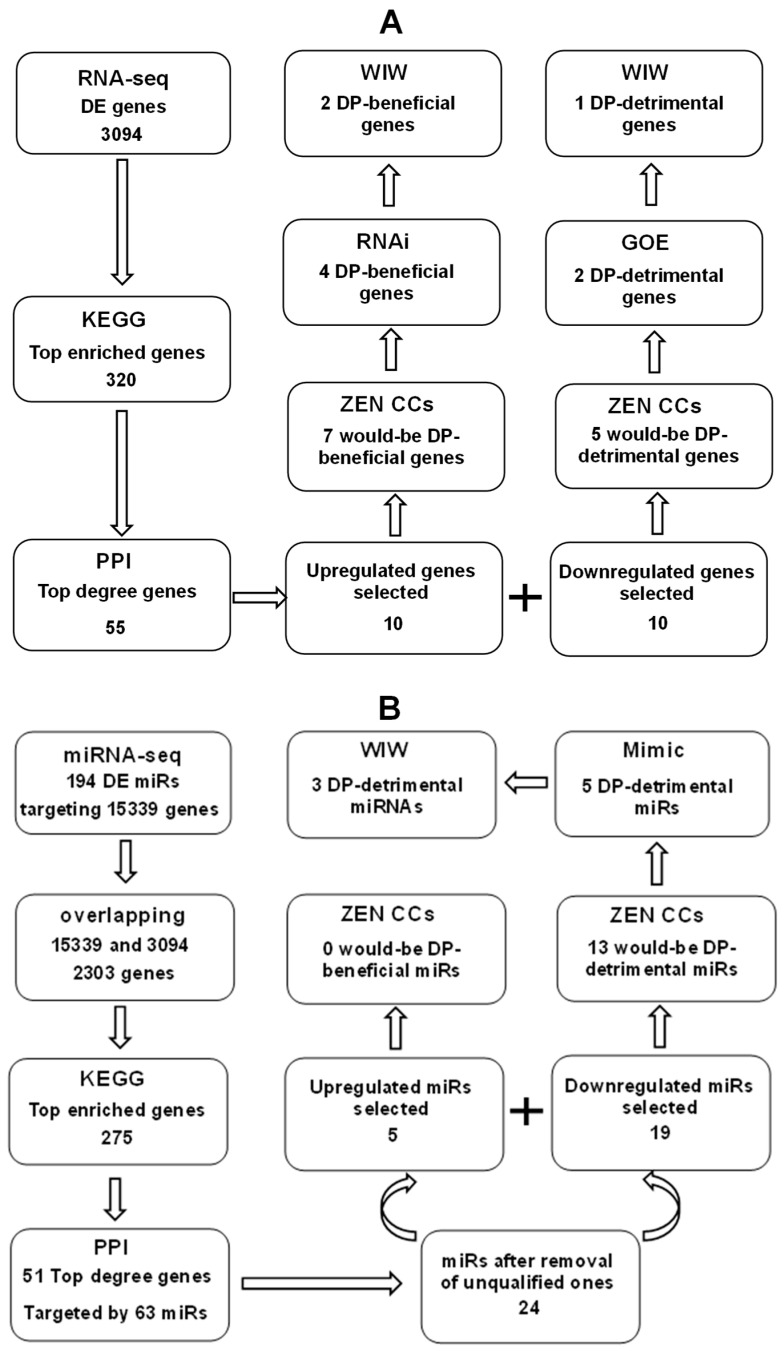
Diagrams showing the procedures for identification and validation of DP-regulating genes (**A**) and DP-regulating miRNAs (**B**). Please refer to the text in the discussion section for detailed explanations.

Our RNA-seq analysis using in vitro CC models identified 3094 DE genes (Figure 9A). Following KEGG pathway analysis of these 3094 DE genes, we obtained 320 top-enriched genes, and subsequent protein-protein interaction (PPI) analysis yielded 55 top-degree genes. From these, 20 genes (10 upregulated and 10 downregulated) were selected based on their FC values. Using in vivo CC models, we validated seven genes as potentially beneficial for DP and five genes as potentially detrimental among the top-degree genes. RNAi and GOE experiments functionally validated four DP-beneficial and two DP-detrimental genes, respectively. Finally, WIW activation and embryo culture confirmed two DP-beneficial and one DP-detrimental gene. Our miRNA-seq analysis identified 194 DE miRNAs targeting 15,339 genes (Figure 9B). An overlap between the 15,339 targeted genes and the 3094 DE genes resulted in 2303 genes. KEGG analysis of these 2303 genes yielded 275 top-enriched genes, and subsequent PPI analysis identified 51 top-degree genes. These 51 top-degree genes were targeted by 63 miRNAs, from which we selected five upregulated (potentially DP-beneficial) and 19 downregulated (potentially DP-detrimental) miRNAs based on fold change values after excluding unqualified candidates. None of the five potentially DP-beneficial miRNAs were validated, but 13 of the 19 potentially DP-detrimental miRNAs were validated using in vivo CC models. Transfection of COCs with miRNA mimics functionally validated five DP-detrimental miRNAs. Finally, WIW activation and embryo culture confirmed three DP-detrimental miRNAs.

In this study, we conducted two KEGG analyses separately using the DE genes and the miRNA-targeted genes. Among the top 10 enriched pathways, 8 were commonly enriched by both the DE genes and the miRNA-targeted genes (Table 3, Exp 1, and Exp 2), indicating a high degree of similarity between the results of these two analyses. When considering the KEGG analysis results from our previous studies (Table 3, Exp 3, and Exp 4), 5 out of the top 10 enriched pathways were consistently enriched across all four experiments. Given that our prior studies focused on CCs-expressed genes and/or miRNAs regulating cumulus expansion and/or CC apoptosis, whereas the current study investigates genes and miRNAs regulating oocyte DP, the common enrichment of 5 KEGG pathways across the four experiments (1) implies that CCs-expressed genes and miRNAs predominantly regulate oocyte DP indirectly through their effects on cumulus expansion and/or CC apoptosis, and (2) further validates that cumulus expansion, CC apoptosis, and CCs-expressed genes/miRNAs can serve as reliable markers for the DP of oocytes and embryos. This conclusion is supported by our findings that oocytes with impaired DP exhibit compromised cumulus expansion but increased CC apoptosis following in vitro maturation with PMS. Consistent with this observation, Zhang et al. [34] reported impaired cumulus expansion and elevated CC apoptosis in in vivo matured oocytes from ZEN-treated mice, which also exhibited impaired DP in this study. Additionally, in bovine COCs, dye transfer from cumulus cells to the oocyte progressively decreased from 0 to 9 h of IVM culture, after which oocyte-cumulus cell gap junction communication was entirely lost [43].

It is reported that the essence of cumulus expansion is the formation of extracellular matrix (ECM) [44], and that ECM is also involved in the regulation of apoptosis [45]. It is frequently found that the pathway of ‘protein digestion and absorption’ is associated with the ‘ECM–receptor interaction’ and the ECM organization pathways [46,47]. The major ECM components, such as ‘proteoglycans’ [44], played important roles in both ‘axon guidance’ [48,49] and cumulus expansion [50], and can inhibit CC apoptosis in granulosa cells [51]. Several molecules in the ‘axon guidance’ pathway are regulated by p53 [52], which induces apoptosis [53]. The ‘focal adhesion’ kinase promoted cumulus expansion [54] while decreasing apoptosis in colon cancer cells [55]. ‘Pathways in cancer’ has relations with apoptosis [56,57], and this pathway includes the transforming growth factor-β signaling that contributes to cumulus expansion [38,58]. Inosine alleviated diabetic peripheral neuropathy with reduced ‘AGE/RAGE’ axis and oxidative stress [59]. When the ‘PI3K/AKT pathway’ was activated, CC apoptosis was inhibited [28,60] and cumulus expansion was facilitated [61]. As for the ‘fluid shear stress and atherosclerosis’ pathway, hemodynamic shear stress may modify ECM of blood vessels [62], and oscillating shear stress can induce endothelial oxidative stress, mitochondrial dysfunction, and metabolic abnormalities [63].

In this study, our RNAi/GOE validated Spp1, Fn1, Sdc1, and Ngf as DP-beneficial genes, while Fos and Jun as DP-detrimental genes. By using the WIW activation and embryo culture system, we validated Spp1 and Ngf as DP-beneficial and Jun as DP-detrimental genes. Our KEGG pathway analysis indicated that Spp1 was enriched in the ‘ECM-receptor interaction’, ‘focal adhesion’, and ‘PI3K-Akt signaling pathway’, which were significantly involved in regulation of cumulus expansion and CC apoptosis. A low expression level of Spp1 was considered as one of the causal factors for the suboptimal development of cultured oocyte-and-granulosa cell complexes [64]. Treatment of ovine oocytes with ZEN inhibited SPP1 expression and in vitro maturation [65]. Ngf was enriched in the PI3K-Akt signaling pathway. NGF activates the PI3K and MAPK signaling pathways to promote cell survival and proliferation during folliculogenesis [66]. Supplementation with NGF promoted ovine in vitro oocyte maturation and early embryo development [67]. Fn1 was enriched in ‘ECM-receptor interaction’, ‘Pathways in cancer’, ‘Focal adhesion’, ‘AGE-RAGE SP in diabetic complications’, ‘Proteoglycans in cancer’, and ‘PI3K-Akt signaling pathway’. It was shown that FN1 improved pig oocyte maturation by promoting activation of the PI3K signaling [68]. FN1 was downregulated in CCs of infertile polycystic ovary syndrome women [69]. Sdc1 was enriched in the ‘ECM-receptor interaction’, ‘Fluid shear stress and atherosclerosis’ and ‘Proteoglycans in cancer’ pathways. Zhang et al. [34] observed that Sdc1 promoted cumulus expansion while inhibiting CC apoptosis.

In this study, among the DP-detrimental genes, Jun was enriched in ‘pathways in cancer’, ‘focal adhesion’, ‘fluid shear stress and atherosclerosis’ and ‘AGE-RAGE SP in diabetic complications’, and Fos was enriched in ‘pathways in cancer’ and ‘fluid shear stress and atherosclerosis’ pathways. It has been reported that treatment of aged mice with growth hormone ameliorated the age-associated depletion of ovarian reserve and decline of oocyte quality via inhibiting the activation of Fos and Jun signaling [70]. The 9-cis retinoic acid inhibits CC apoptosis with downregulated expression of c-Jun N-terminal kinase (JNK), c-jun and c-fos [71]. Furthermore, the LEA proteins improved the developmental potential of cryopreserved human oocytes by downregulating FOS and reducing oxidative stress [72].

In this study, our miRNA function analysis with mimics validated 5 DP-detrimental miRNAs, including miR-7686-5p, 133a-3p, novel-239, novel-193, and miR-339-5p, and our WIW further validated miR-7686-5p, novel-239, and miR-339-5p as detrimental miRNAs. It was reported that miR-7686-5p was upregulated in the testes of mice exposed to a mixture of environmental endocrine disruptors [73]. Han et al. [74] observed that overexpression of miR-339-5p significantly decreased porcine oocyte rates of maturation and blastocyst formation. Both novel-miR-239 and novel-miR-193 were discovered for the first time in the present study, and their effects have not been reported. The miR-133a-3p inhibited proliferation while promoting apoptosis of the intestinal epithelial cells [75] and the esophageal squamous cell carcinoma [76].

## 5. Conclusions

Urgent research is required to identify and validate CCs-expressed genes and miRNAs whose expression levels are directly correlated with oocyte DP for use as biomarkers in selecting competent oocytes and embryos. In this study, by performing mRNA/miRNA sequencing and functional analyses using both in vitro and in vivo CC models derived from COCs with significantly differing DP, we identified and validated Spp1, Fn1, Sdc1, and Ngf as DP-beneficial genes; Fos and Jun as DP-detrimental genes; and miR-7686-5p, miR-133a-3p, novel-miR-239, novel-miR-193, and miR-339-5p as DP-detrimental miRNAs. Using a WIW activation/embryo culture system that enables tracking the COC origin of CCs and embryos, we further validated Spp1 and Ngf as DP-beneficial genes, Jun as the DP-detrimental gene, and miR-7686-5p, novel-miR-239, and miR-339-5p as DP-detrimental miRNAs. Furthermore, the close similarities in top KEGG pathways across four distinct experiments suggest that CCs-expressed genes and miRNAs predominantly regulate oocyte DP indirectly through their effects on cumulus expansion and/or CC apoptosis. The newly identified genes and miRNAs can serve as markers for selecting competent oocytes and embryos, and the data generated will contribute to elucidating the mechanisms underlying oocyte maturation.

## Figures and Tables

**Table 1:** Sequences of primers used in RT-qPCR for mRNAs.

mRNAs	Forward	Reverse
β-actin	AGTGTGACGTTGACATCCGT	GCAGCTCAGTAACAGTCCGC
*Stella*	AGGGTCCGCACTTTGTTGTC	GGCTCACTGTCCCGTTCAAA
*Mater*	TCAAGAACTGGAACTAGTGGAC	GCTTGGTTGTTGTGATCATACA
*Zar1*	GACGCCTCGGTGCAGTGTTC	CACAGAAGGTCACGGACGAGAAC
*Mapk8*	AAACTGTTCCCCGATGTGCTT	CGTTGATGTATGGGTGCTGGA
*Bcl2*	GAGCGTCAACAGGGAGATG	GGGCCATATAGTTCCACAAAGG
*Bax*	TGCAGAGGATGATTGCTGAC	GATCAGCTCGGGCACTTTAG
*Foxj2*	TCAGCAAAGATGAGGCAGCG	ACCGATGCCAGCGTTCTTGTA
*Shp1*	GGGCTAGACTGTGACATTGATA	TTTCTTCTTGGTCGTTTCGATG
*Fn1*	ATCATAGTGGAGGCACTGCAGAA	GGTCAAAGCATGAGTCATCTGTAGG
*Star*	ACTCACTTGGCTGCTCAGTATTG	CAGGTGGTTGGCGAACTCTAT
*Itgb3*	GGCGTTGTTGTTGGAGAGTC	CTTCAGGTTACATCGGGGTGA
*t5a*	GACGCTAGAGAAAGGGAACGAATC	GCCAGACACTCCATGACACTTAC
*c4*	AGGGCAGCAACATCTTTGAGAGAA	CACCAGCAGCAGGATCAGGAA
*Scd1*	TACACCTGCCTCTTCGGGATT	CAGCCGTGCCTTGTAAGTTCT
*Hspg2*	ATCTGTAACGCCACCAACTCT	GCCGAGGTATGTTCTGGAATTATG
*Itga7*	TACCTCATCCTCAGCACCTCT	GACAGTGGCAATTCAATGAAGACA
*Col4a2*	CAGCCTGGTGTACTCGGTCTTC	TTGGTCGCCTTTGGGTCCTTT
*Stc2*	AGTTTGTGACCCTGGCTTTGG	CTGGATCTCCGCTGTGTTCTG
*Itga6*	TCTCGTTCTTCGTTCCAGGTTGT	AGCAGCAGCGGTGACATCTAT
*Pik3cg*	TGACAGGCACAACGACAACATT	TTAGGACGAAGGGCACTCTCTC
*Cxcl12*	TGCATCAGTGACGGTAAACCA	TTCTTCAGCCGTGCAACAATC
*Itga9*	CCATCAACATCACAGCACCTCAG	TCAGCCGTCAGATTGTAGTTCAGA
*Thbs1*	GACTATGACAAGGACGGGATTGG	ACTGGGCTGGGTTGTAATGGAA
*Ar*	TTCCTGGATGGGACTGATGGTA	CGAGACTTGTGCATGCGGTA
*Bmp4*	GGGATCTTTACCGGCTCCAG	GCTGCTGAGGTTGAAGAGGA
*Spp1*	TACGACCATGAGATTGGCAGTGA	TATAGGATCTGGGTGCAGGCTGTAA
*Mmp2*	CAGGGCACCTCCTACAACAG	CAGTGGACATAGCGGTCTCG
*Col18a1*	GCCAGAAAGGCAGTGTTGGTG	GAATCCAGCAGCAAATCCTGG
*Ngf*	GCGTTTTTGATCGGCGTACA	AGGGCTGTGTCAAGGGAATG
*Nrp1*	GGAGGAATGTTCTGTCGCTATG	GCACACTGTAGTTGGCTGAG
*Agt*	TCTCCTTTACCACAACAAGAGCA	CTTCTCATTCACAGGGGAGGT
*Vcam1*	TCAGGAAATGCCACCCTCAC	CAGCACACGTCAGAACAACC
*Edn1*	TCTGCCACCTGGACATCATCT	AACGCTTGGACCTGGAAGAAC
*Fos*	ACCGTGTCAGGAGGCAGA	GCAGCCATCTTATTCCGTTCC
*Icam1*	GTGGGTCGAAGGTGGTTCTT	CCGAGGACCATACAGCACG
*Igf1*	CAGTTCGTGTGTGGACCGAG	AGTGGGGCACAGTACATCTC
*Csf1*	CGCTGCCCTTCTTCGACA	AGGCAATCTGGCATGAAGTCT
*Kitl*	TGATAACCCTCAACTATGTCGCC	TGTCCAGAAGAGTAGTCAAGCTG
*Jun*	ACGACCTTCTACGACGATGC	GCCAGGTTCAAGGTCATGCT

**Table 2:** Sequences of primers used in RT-qPCR for miRNAs.

miRNAs	Forward	Reverse
miR-140-3p	TACCACAGGGTAGAACCACGG	mRQ 3′ Primer from TAKARA miRNA RT-qPCR kit (TaKaRa Bio Inc., Kusatsu, Japan)
miR-149-5p	TCTGGCTCCGTGTCTTCACTCCC	
miR-365-2-5p	GGACTTTCAGGGGCAGCTAAA	
miR-199a-5p	TCTCCCAGTGTTCAGACTACCT	
miR-7686-5p	GACCTGGGGCTGGGCAAAA	
miR-133a-3p	CTCCCCTTGAACCAGCTGAAA	
novel-miR-248	GCTGGCCTTGAACTCACAGAA	
novel-miR-239	AGTTCCTTGGCTGTGTCTGAG	
miR-351-3p	TTCAAGATGCGCCTGGGAAC	
miR-145a-5p	CAGTTTTCCCAGGAATCCCT	
miR-3473a	GGAGAGATGGCTCAGCAAAA	
miR-667-5p	GTGGAGCAGTGAGCACGAAA	
miR-351-5p	TCCCTGAGGAGCCCTTTGAGCCTG	
novel-miR-28	CGAGATCGTGGGTTCGAGTC	
novel-miR-168	CGAGATCGTGGGTTCGAGTC	
novel-miR-340	CCTGAGTGTGTGTGTGTGTATTA	
novel-miR-193	TGGACAACCCAGGAGGTCAA	
novel-miR-85	GGGAATTGAACTCAGGACCTAA	
miR-615-5p	CCCGGTGCTCGGATCAAAA	
miR-7686-3p	CTCGGGGCACTGTAAGAGAA	
miR-700-3p	GGGAACCGAGTCCACCAAA	
miR-128-1-5p	GGCCGTAGCACTGTCTGAAA	
miR-339-5p	CCTCCAGGAGCTCACGAAAA	
miR-326-3p	TGGGCCCTTCCTCCAGTAAA	
miR-467d-3p	GCATACATACACACACCTACACAA	
U6	GGAACGATACAGAGAAGATTAGC	TGGAACGCTTCACGAATTTGCG

**Table 3:** The top 10 KEGG pathways enriched by DE or miR-targeted genes of CCs in four different experiments.

*KEGG Pathways*	*Exp 1*	*Exp 2*	*Exp 3*	*Exp 4*
**ECM-receptor interaction # $**	1 *	**1**	5	6
**Pathways in cancer**	**2**	**5**	3	7
**Axon guidance**	**3**	**2**		1
**Focal adhesion**	**4**	**3**	2	3
DNA replication	5			
Fluid shear stress and atherosclerosis	6		6	
**Protein digestion and absorption**	**7**	**4**	1	9
**AGE-RAGE SP in diabetic complications**	**8**	**6**		
**Proteoglycans in cancer**	**9**	**7**		4
**PI3K-Akt signaling pathway**	**10**	**8**	4	5
Metabolic pathway			8	
Cushing syndrome			9	
EGFR tyrosine kinase inhibitor resistance			10	
Wnt signaling pathway		9	7	
Regulation of actin cytoskeleton		10		
Rap1 signaling				2
Regulation lipolysis adipocytes				8
Ras signaling				10

Exp 1: KEGG analysis of the DE genes in this study. Exp 2: KEGG analysis of the miR-targeted genes in this study. Exp 3: Zhang et al. (2024) [34], KEGG analysis of the DE genes. Exp 4: Han et al. (2022) [25], KEGG analysis of genes from overlapping between DE genes and miR-targeted genes. * *p* value raking number of pathways in the same experiment (column). # Bold pathways were enriched by both the DE and the miR-targeted genes in Exp 1 and Exp 2. $ Pathways in shaded boxes were commonly enriched in all the four experiments.

## Data Availability

The data presented in this study are openly available in [https://www.ncbi.nlm.nih.gov/] at [https://www.ncbi.nlm.nih.gov/], reference number [GSE291430].
